# Safety Evaluation of the Polyherbal Formulation NawaTab: Acute and Subacute Oral Toxicity Studies in Rats

**DOI:** 10.1155/2023/9413458

**Published:** 2023-07-24

**Authors:** Apichaya Niyomchan, Wasapon Chatgat, Bodin Chatawatee, Thaweeporn Keereekoch, Acharaporn Issuriya, Patcharawalai Jaisamut, Sasitorn Chusri, Nongluk Kunworarath

**Affiliations:** ^1^Department of Anatomy, Faculty of Medicine Siriraj Hospital, Mahidol University, Bangkok 10700, Thailand; ^2^Faculty of Traditional Thai Medicine, Prince of Songkla University, Hat Yai, Songkhla 90110, Thailand; ^3^Traditional Thai Medical Hospital, Faculty of Traditional Thai Medicine, Prince of Songkla University, Hat Yai, Songkhla 90110, Thailand; ^4^Traditional Thai Medical Research and Innovation Center, Faculty of Traditional Thai Medicine, Prince of Songkla University, Hat Yai, Songkhla 90110, Thailand; ^5^Division of Health and Applied Sciences, Faculty of Science, Prince of Songkla University, Hat Yai, Songkhla 90110, Thailand; ^6^School of Health Science, Mae Fah Luang University, Muang, Chiang Rai 57100, Thailand

## Abstract

NawaTab is a tablet formulation developed from the Nawametho polyherbal formula used in Surat Thani province, Southern Thailand, for the treatment of hyperlipidemia. This study aims at evaluating the acute and subacute toxicity of NawaTab in rats. In the acute toxicity study, NawaTab was evaluated in female rats following the OECD Guideline No. 423. In the subacute toxicity study, NawaTab was tested in both male and female rats following the OECD Guideline No. 407. In the acute toxicity study, no lethal effects or toxic signs were observed during the duration of the study. In the subacute toxicity study, there was no mortality and no abnormality in clinical signs, body weight, food consumption, relative organ weight, and hematological parameters of NawaTab-treated rats. Significantly increased water consumption by male rats (500 mg/kg BW) and female rats (250, 500, and 1000 mg/kg BW) was observed. In addition, globulin and total cholesterol of female rats (1000 mg/kg BW) significantly increased. These alterations were within normal physiological ranges. Moreover, necropsy and histopathological findings of NawaTab-treated rats demonstrated no obvious alterations attributable to NawaTab administration. The present study revealed that NawaTab has no significant acute oral toxicological effects. The lethal dose with a 50% mortality rate (LD_50_) was higher than 5000 mg/kg BW in rats. The subacute oral administration of NawaTab for 28 days did not have any major toxicological effects. Based on this study, NawaTab could be safe to use with caution pending its chronic toxicity study.

## 1. Introduction

Traditional herbal medicines have been used for a long time to prevent and/or treat a variety of diseases. In addition, their uses are widely accepted since they are recognized as nontoxic, have fewer side effects, and are affordable when compared to modern chemical drugs [1]. However, adverse effects of traditional herbal medicines may occur in clinical practice [1, 2]. Therefore, a safety evaluation of herbal formulations is necessary to identify their possible toxic effects and ensure their quality and safety.

Nawametho is an herbal recipe combining 9 herbal medicines: *Aegle marmelos* (L.) Correa, *Carthamus tinctorius* Linn., *Hibiscus sabdariffa* L., *Piper longum* L., *Piper nigrum* L., *Phyllanthus emblica* L., *Zingiber officinale* Roscoe., *Terminalia bellirica* (Gaertn.) Roxb., and *Terminalia chebula*. These components of Nawametho are commonly used in traditional medicine in many countries, even in Thailand. Moreover, these herbal components in Nawametho contain antioxidant activities which protect the cells against free radicals [3–6]. Furthermore, research studies confirm their antioxidant properties against metabolic diseases, especially in hyperlipidemia. A decrease in the levels of total cholesterol, triglycerides, and low-density lipoproteins has been established in animal and human studies [4, 5, 7–17]. Nawametho decoction has been used in Ban-ta-khun hospital in Surat Thani province, in the southern peninsular part of Thailand, to treat hyperlipidemia. However, there is no information about its toxicity. In addition, NawaTab, an oral tablet dosage form of Nawametho decoction, has been developed under a quality control system to increase product quality and stability and to improve patient compliance for improved treatment outcomes. Therefore, it is necessary to assess the safety of NawaTab.

As part of a safety evaluation of NawaTab, acute and subacute oral toxicity studies were conducted to investigate the potential toxicity after a single or 28-day repeated daily oral administration of NawaTab in Wistar rats. The present study was carried out following the guidelines of the Organization for Economic Cooperation and Development [18, 19].

## 2. Materials and Methods

### 2.1. Preparation of NawaTab

The herbal ingredients for NawaTab were qualified following the Thai herbal pharmacopoeia [20, 21]. The specification values for crude drugs are shown in the previous study [22].

NawaTab is a tablet formulation developed from the Nawametho herbal recipe. Briefly, the tablet was prepared by the dry granulation technique from the dried extract of Nawametho decoction, diluent, lubricant, disintegrant, and antiadherent. First, the extract, diluent, and lubricant were weighed and mixed in an appropriate mixer for making the slug. The slug was passed through a mesh screen and dried in an incubator. To get dry granules, the slug was reduced in size by passing it through a mesh screen again. Disintegrant and antiadherent were added to the mixture. Lastly, the dry granules were compressed using round punches and flat-face dried in a single-punch tablet press. The methods of NawaTab preparation and quality control following The United States Pharmacopeia Convention [23] and British Pharmacopoeia Commission [24] were mentioned in petty patent number 1903002586.

### 2.2. Animals

Male and female Wistar rats (aged 4–7 weeks) were used for acute and subacute toxicity studies. Five rats were housed per cage with free access to a normal diet and water. The rats were kept in a temperature-controlled room (22 ± 2°C), with a 12 h light/dark cycle and a relative humidity of 55 ± 10%. The animals were acclimatized to the laboratory conditions for one week prior to the experiments. All procedures were approved by the Animal Ethical Committee of Prince of Songkla University (MOE 0521.11/876).

### 2.3. Acute Toxicity Study

The acute oral toxicity of NawaTab was evaluated in female rats following the OECD Guideline No. 423 [18]. The NawaTab was dissolved in distilled water. All animals were fasted overnight prior to dosing and for 3 h after treatment. For the starting dose, three rats were given single-dose which was orally administered with NawaTab at a dose of 2000 mg/kg BW. These animals were observed for clinical signs or mortality periodically during the first 24 h (0.5, 1, 2, 3, 4, and 24 h). When no mortality or apparent signs of toxicity were observed within 24 h posttreatment, the procedures were repeated in another three rats using 2000 mg/kg BW of NawaTab. A higher dose group (5000 mg/kg BW) was tested using the same procedure when all animals in the starting dose treatment group survived. Thereafter, animals were weighed weekly, and observations were made once daily for 14 days. At the end of the experiment, all animals were sacrificed and subjected to gross necropsy.

### 2.4. Subacute Toxicity Study

The subacute oral toxicity of NawaTab was tested in both male and female rats following the OECD Guideline No. 407 [19]. The rats were randomly divided into four treatment groups (5 rats/sex/group). Rats were orally administered either distilled water (control) or NawaTab (250, 500, and 1000 mg/kg BW) every day for 28 days. Body weight, food consumption, and water consumption were recorded daily. Animals were observed daily with a focus on mortality, clinical signs of toxicity, changes in general behavior, skin, eyes, fur, mucous membranes, unusual respiration patterns, and somatomotor activity. Attention was directed to monitoring sleep, diarrhea, tremors, convulsions, lethargy, and coma. After 28 days, all animals were fasted overnight and anesthetized by an intraperitoneal injection of pentobarbitone sodium. Blood samples were collected via cardiac puncture and used for hematology and clinical biochemistry analyses. The hematologic parameters recorded were white blood cell (WBC), neutrophil (NE), lymphocyte (LY), monocyte (MO), eosinophil (EO), basophil (BA), red blood cell (RBC), hemoglobin (HGB), hematocrit (HCT), platelet count (PLT), mean corpuscular volume (MCV), mean corpuscular hemoglobin (MCH), mean corpuscular hemoglobin concentration (MCHC), and red blood cell distribution width (RDW). Clinical biochemistry values were alkaline phosphatase (ALP), alanine aminotransferase (ALT), aspartate aminotransferase (AST), total bilirubin (T-Bil), direct bilirubin (D-Bil), total protein (TP), globulin (Glo), albumin (Alb), blood urea nitrogen (BUN), creatinine (Cre), triglyceride (TG), and total cholesterol (TC). After taking the blood sample, the internal organs were examined for macroscopic and microscopic abnormalities. The organs such as the heart, liver, lungs, spleen, kidneys, and reproductive organs were weighed, and the relative weight of each organ was calculated. After weighing, fragments of selected organs, namely, the liver, kidney, and lung, were immediately fixed in 10% neutral buffered formalin, dehydrated with alcohol, and embedded in paraffin wax. Serial sections were cut with a rotatory microtome (Leica RM2035, Nussloch, Germany) to 6 *µ*m thickness. After deparaffinization, the sections were stained with hematoxylin-eosin (H&E). Light micrographs of multiple tissue sections from each organ in all groups were taken using an Olympus Bx43 microscope and DG73 digital camera attached to the CellSens Standard Software (Olympus Optical Co. Ltd., Tokyo, Japan).

### 2.5. Statistical Analysis

The data are expressed as the mean ± standard error of the mean (SEM). Each dataset was analysed by training a statistical decision tree. The data were initially analysed for homogeneity of variance using Levene's test, followed by the Shapiro–Wilk test for normality. If both Levene's test and the Shapiro–Wilk test were not significant, the data were assessed by analysis of variance (ANOVA) followed by Dunnett's test. If either Levene's test or the Shapiro–Wilk test was significant, then the data were analysed using the Kruskal–Wallis nonparametric ANOVA followed by Dunn's test. Statistical significance was defined as *p* < 0.05. All calculations were performed using the SPSS software package for Windows.

## 3. Results

### 3.1. Acute Toxicity Study

Oral administration of NawaTab at doses of 2000 and 5000 mg/kg BW produced no mortality or toxicity during the 14 days of the experiment. After 14 days of NawaTab treatments, no abnormal body weight gain or food or water consumption was observed. In addition, there were no significant differences in body weight gain or in food and water consumption between rats treated with NawaTab 2000 and 5000 mg/kg BW. No behavioral changes were observed in the rats. Moreover, macroscopic and microscopic analysis of the rat organs did not show any abnormal findings to indicate toxicity.

### 3.2. Subacute Toxicity Study

**Figure 1:**
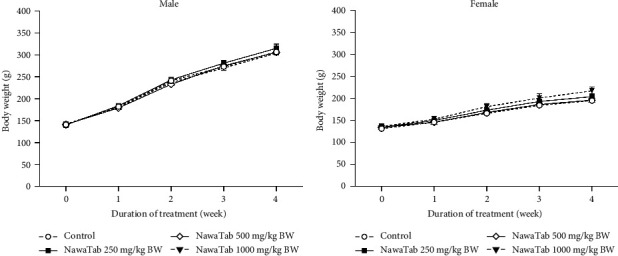
Body weight of male (a) and female (b) NawaTab-treated rats in the subacute toxicity study.

No behavioral changes, clinical signs, and mortality were observed in the rats during the 28 days of treatment. A significant increase in water consumption presented in NawaTab-treated female rats at doses 250, 500, and 1000 mg/kg BW and in NawaTab-treated male rats only at dose 500 mg/kg BW, when compared to the respective control groups (Table 1). However, food consumption, body weight, and percentage of body weight gain were not affected by daily NawaTab oral administration (Figure 1 and Table 1). These observations suggest that NawaTab did not alter the growth of rats.

As shown in Table 2, clinical biochemistry analysis revealed an elevation of globulin and total cholesterol in female rats treated with NawaTab at 1000 mg/kg BW. However, no increase in globulin or total cholesterol was observed in the other treatment groups (250 and 500 mg/kg BW). Other clinical biochemistry parameters were not changed after 28 days of NawaTab treatment (250, 500, and 1000 mg/kg BW). In addition, there were no significant effects on hematological parameters in rats after 28 days of NawaTab treatment (Table 3).

**Figure 2:**
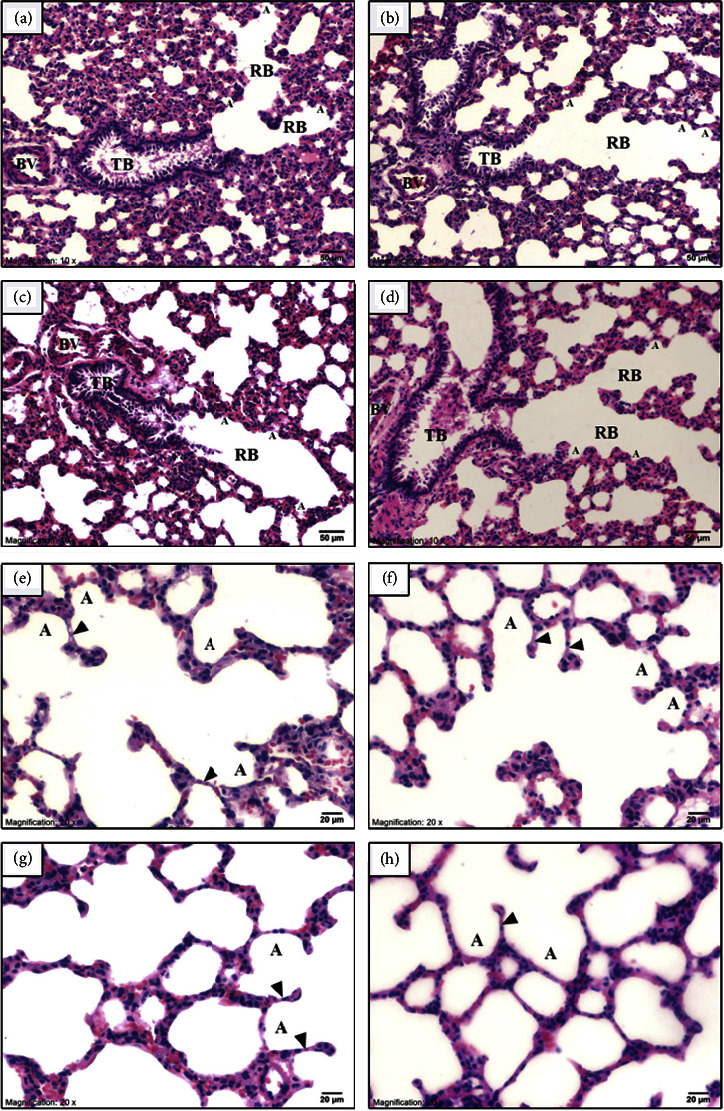
Light micrographs of lung tissues in control rats (a) and rats treated with 250 (b), 500 (c), and 1000 (d) mg/kg BW of NawaTab showing the distal part of the conducting portion and respiratory portion. Light micrographs of lung tissues showing normal structure of alveoli (A) with thin interalveolar septum (arrowhead) in all experimental groups, including control (e), 250 (f), 500 (g), and 1000 (h) mg/kg BW of NawaTab. The terminal bronchiole (TB) contained folded columnar epithelial cells that were surrounded by a smooth muscle layer. The respiratory bronchioles (RB) continuing from the terminal bronchioles, had some alveoli (A) along their walls. BV: blood vessel.

**Figure 3:**
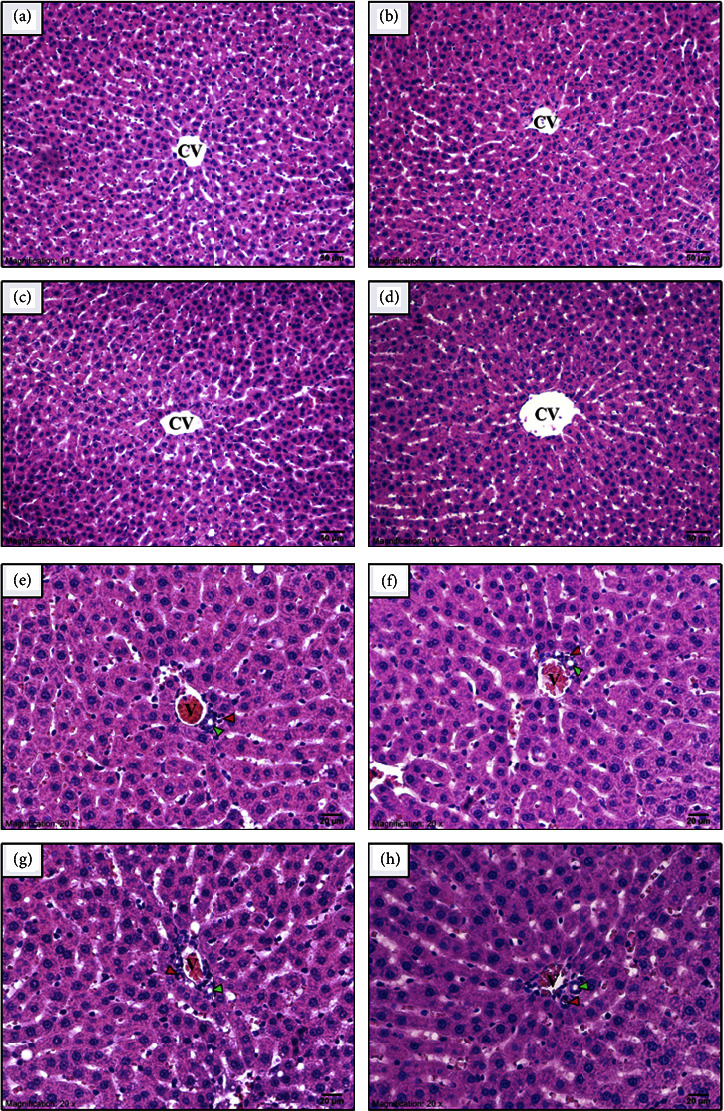
Light micrographs of liver tissues showing the classic hepatic lobule including the middle of the central vein (CV), hepatocytes arranged in radiating cords from the central vein, and sinusoidal spaces between the cords. Normal hepatic morphology was found in all experimental groups. (a) Control, (b) 250, (c) 500, and (d) 1000 mg/kg BW of NawaTab. Light micrographs of liver tissue showing the portal area and its structures, including the interlobular branches of the bile duct (green arrowhead), hepatic artery (red arrowhead), and portal vein (V) surrounded by hepatocytes. There were no differences in portal structures between any of the experimental groups. (e) Control, (f) 250, (g) 500, and (h) 1000 mg/kg BW of NawaTab.

**Figure 4:**
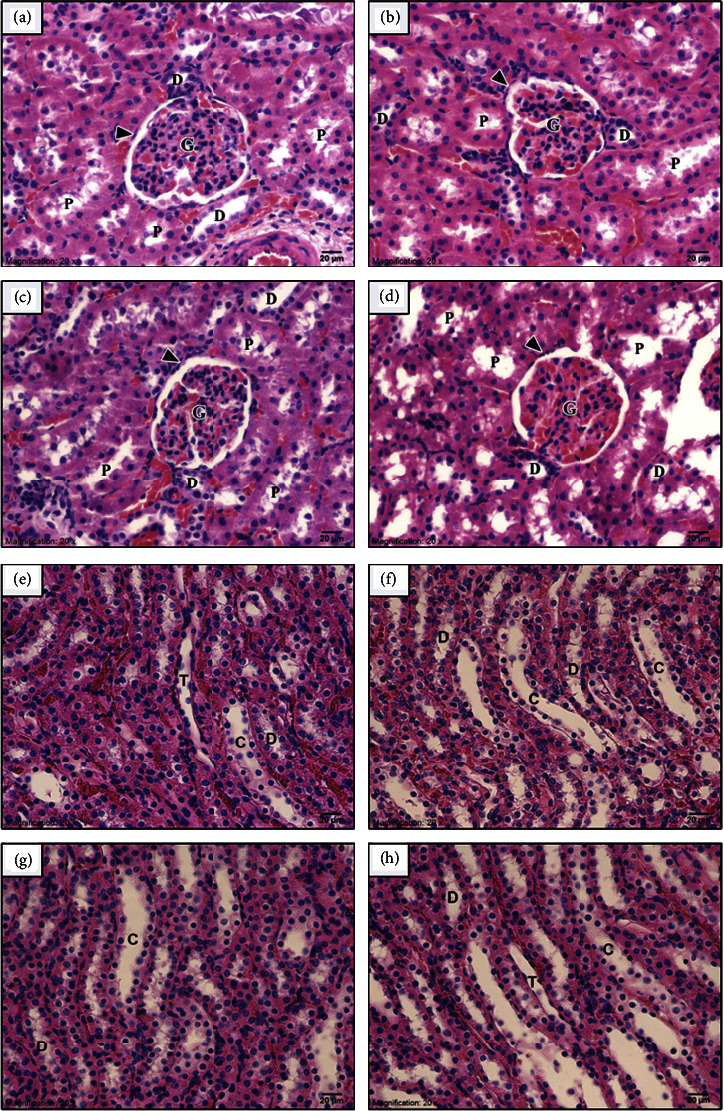
Light micrographs of renal cortex showing the normal glomerulus (G) enveloped by Bowman's capsule (arrowhead), proximal (P), and distal (D) tubules for all experimental groups. (a) Control, (b) 250, (c) 500, and (d) 1000 mg/kg BW of NawaTab. Light micrographs of the renal medulla showing normal structure of renal tubules including distal tubules (D), thin segment of Henle's loop (T), and collecting tubules (C) for all experimental groups. (e) Control, (f) 250, (g) 500, and (h) 1000 mg/kg BW of NawaTab.

No abnormal findings were observed in external and internal gross pathology. In addition, no significant changes were observed in the relative organ weights of NawaTab-treated rats (Table 4). The macroscopic appearance of the lung, liver, and kidney in both the control and NawaTab-treated groups showed regular smooth surfaces and soft consistency. Conventional light microscopy images after H&E staining of these organs in control and NawaTab-treated rats are shown in Figures 2–4. No abnormal morphology and histological lesions were found in these organs of NawaTab-treated rats at any of the dose levels tested, when compared with those in the control rats.

Pulmonary parenchyma of the rats' lung consisted of conducting and respiratory portions (Figure 2). The distal part of the conducting portion was the terminal bronchiole, which divided into respiratory bronchioles. Their walls were interrupted by the opening of alveoli. Control rats showed normal lung architecture including folded columnar epithelial cells of the terminal bronchiole, normal pulmonary vessels, interstitial tissue, thin interalveolar septa, and an alveolar sac. There were no pulmonary histological changes in the rats that received 250, 500, and 1000 mg/kg BW of NawaTab (Figures 2(a)–2(d)).

High magnification of the alveolar system revealed normal characteristics in all groups (Figures 2(e)–2(h)). Alveolar epithelium comprised dominantly flat type I pneumocytes and a minority of cuboid type II pneumocytes or surfactant-producing secretory cells. Alveoli were surrounded by dense capillaries and separated from each other by a thin alveolar septum.

Liver sections of the rats for all toxicity studies showed normal histological structures of the liver lobule and portal area, when compared to the control group (Figures 3(a)–3(d)). The hepatic lobule consisted of the central vein at the center, plates or cords of hepatocytes radiating outward from the central vein to the portal triads, and sinusoids between the hepatic plates. Polygonal hepatocytes were arranged in cords. The cytoplasm was stained pink by eosin in H&E staining and was filled with basophilic granules. The spaces between the hepatic cords were sinusoids. The single large blood vessel in the middle of each lobule was the central vein. It was lined with a layer of flattened endothelial cells, which were continuous along the sinusoid.

At the vertices of the lobule were portal areas or portal triads. A cross section of the triad presented three major tubes of interlobular branches of the bile duct, hepatic artery, and portal vein (Figures 3(e)–3(h)). The branch of the bile duct was lined with simple cuboidal epithelium with conspicuous round nuclei. The interlobular branch of the portal vein was irregularly shaped with only endothelial lining, whereas the interlobular branch of the hepatic artery was round or oval-shaped with a thicker wall. These structures were wrapped together by connective tissue. No histopathological changes were observed in the portal triads of the liver tissues for any of the experimental groups.

The subacute administration of NawaTab had no effect on the kidney either. Normal histological features of the glomeruli and renal tubules in the cortical and medullary portions were observed in the kidney tissue of rats for all toxicity studies compared to the control (Figure 4).

The renal cortex was occupied by the renal corpuscles and renal tubules. The renal corpuscle was a relatively spherical structure consisting of a knot of glomerular capillaries surrounded by a double-walled Bowman's capsule including the outer parietal and inner visceral layers of simple squamous epithelium. The proximal tubules were lined by a simple cuboidal epithelium with a brush border on their luminal surface. They exhibited small and uneven lumens. Distal tubules were formed by a single layer of low cuboidal epithelium without a brush border. Their cell lining had deeply basophilic stained nuclei and less acidophilic cytoplasm. They had larger lumens than those of the proximal tubules and were few in number in the cortex (Figures 4(a)–4(d)).

The renal medulla contained thick and thin parts of the loops of Henle, collecting tubules, and collecting ducts. The thick descending part of Henle's loop continued to the proximal tubule in the cortex, while the thick ascending part was similar to the distal tubule in the cortex. The cell lining of the collecting tubules was cuboidal in type and became columnar-shaped in the collecting duct close to the renal papilla. The boundary of the cells forming the collecting tubules was clear, and the cytoplasm was pale-stained when compared with the cells of the proximal and distal tubules (Figures 4(e)–4(h)).

## 4. Discussion

Generally, the use of herbal medicines is considered safe because of the accumulated experiences over a long history of usage. Unlike chemical drugs, herbal medicines are not subject to toxicity tests prior to clinical application. Due to the increasing use of herbal medicines worldwide, evaluations of their efficacy and safety are necessary to ensure their quality and safety [1, 25]. Nawametho is a polyherbal recipe used to treat hyperlipidemia. Recently, Nawametho has demonstrated its effectiveness in clinical use [22]. NawaTab, a tablet formulation developed from Nawametho decoction, has been developed to increase product quality and stability and to improve patient compliance for improved treatment outcomes. Although the previous study showed antihyperlipidemic effects of NawaTab in a high-fat diet-induced hyperlipidemic rat model [26], its safety has not been evaluated. Therefore, this study aims to evaluate the acute and subacute toxicity of NawaTab in Wistar rats.

In the acute toxicity study, a single oral administration of NawaTab in rats at 2000 and 5000 mg/kg BW had no effect on mortality, body weight, and clinical signs. In addition, no abnormalities were detected upon morphohistological examination of the internal organs of NawaTab-treated rats. Based on these results, the approximate LD_50_ value for the oral dose of NawaTab was higher than 5000 mg/kg BW in rats. According to the Globally Harmonized System (GHS) toxicity classification, substances with oral LD_50_ in the range 2000–5000 mg/kg BW have relatively low toxicity [27]. By this classification, the present study has demonstrated that NawaTab is classified in GSH as category 5, which is relatively low acute toxicity.

In the subacute toxicity study, no mortality was observed throughout the experiment. No significant changes were observed in general appearance, behavior, body weight, percentage of body weight gain, and food consumption in any of the NawaTab-treated rats. However, significantly increased water consumption was noted for NawaTab-treated female rats (250, 500, and 1,000 mg/kg BW) but without toxicological significance because this was within the normal consumption levels [28].

Hematology and clinical biochemistry parameters are important markers of the overall health status of animals and can be used to investigate the toxicity of drugs and chemicals. The present study revealed that oral administration for 28 days did not lead to any significant differences in the hematology parameters of NawaTab-treated rats when compared with controls. Significant elevations in the levels of globulin and total cholesterol were found only in female rats treated with NawaTab at a 1000 mg/kg BW daily dose when compared to the respective controls. However, the increased globulin remained within the normal physiological range. In addition, there were no significant alterations in serum liver enzyme (ALP, AST, and ALT) values and other hepatic function-related parameters such as total protein and albumin, indicating that NawaTab did not cause deterioration in liver function. Moreover, the significant increase in total cholesterol is of questionable toxicological significance because it was within the normal biological variation [29]. A change in total cholesterol level is considered to indicate altered lipid metabolism [30], for which the underlying reason must be further investigated. This finding suggests that prolonged treatment with high doses of NawaTab (≥1000 mg/kg BW) should be avoided. However, our previous study showed antihyperlipidemic effects of NawaTab at a dose of 125 mg/kg/day [26], which is a nontoxic dose.

Observation of relative organ weights is crucial in assessing the safety of a drug [31, 32]. There were no significant changes in relative organ weights in NawaTab-treated rats. Since vital functionally crucial organs are often impaired by toxic substances [33], histopathological examinations of the lung, liver, and kidney were further performed to identify potential signs of organ-targeting toxicity. The results revealed normal morphological appearance and histological features in NawaTab-treated rats.

## 5. Conclusion

The present study demonstrated that NawaTab has no acute oral toxicity and that the oral LD_50_ value is higher than 5000 mg/kg BW in female rats. Moreover, NawaTab did not exhibit any major toxicological effects during oral administration daily for 28 days at the tested dose levels. All changes observed in subacute toxicity studies that attained statistical significance were within normal physiological ranges. Based on these results, NawaTab could be safe to use. Further subchronic and chronic toxicity studies should be conducted to ensure safety in long-term use.

## Figures and Tables

**Table 1:** Body weight gain and food and water consumption of NawaTab-treated rats in the subacute toxicity study.

Parameters	Control	NawaTab (mg/kg BW)		
		250	500	1000
*Males*				
Body weight gain (%)	117.8 ± 4.0	125.0 ± 3.0	117.5 ± 5.5	114.4 ± 3.5
Food consumption (g/day)	20.0 ± 0.4	20.8 ± 0.4	20.0 ± 0.5	20.0 ± 0.4
Water consumption (ml/day)	25.9 ± 0.7	27.7 ± 0.7	28.6 ± 0.7^*∗*^	26.2 ± 0.6

*Females*				
Body weight gain (%)	49.1 ± 3.7	52.6 ± 6.1	46.5 ± 3.7	57.2 ± 3.8
Food consumption (g/day)	14.6 ± 0.3	15.8 ± 0.4	14.7 ± 0.5	14.1 ± 0.7
Water consumption (ml/day)	25.4 ± 0.5	29.6 ± 0.6^*∗*^	29.8 ± 0.9^*∗*^	31.1 ± 0.9^*∗*^

Data are expressed as the mean ± S.E.M., *n* = 5 animals/sex/group. ^*∗*^*p* < 0.05, significantly different from the control group.

**Table 2:** Clinical biochemistry parameters of NawaTab-treated rats in the subacute toxicity study.

Parameters	Control	NawaTab (mg/kg BW)		
		250	500	1000
*Males*				
ALP (U/L)	140.8 ± 12.1	152.4 ± 11.3	121.0 ± 7.0	123.8 ± 8.9
ALT (U/L)	33.8 ± 2.5	31.6 ± 2.2	27.6 ± 1.4	28.2 ± 1.8
AST (U/L)	128.6 ± 14.1	134.8 ± 7.5	112.6 ± 14.9	124.4 ± 14.9
T-bil (mg/dL)	<0.15	<0.15	<0.15	<0.15
D-bil (mg/dL)	<0.09	<0.09	<0.09	<0.09
TP (g/dL)	5.62 ± 0.02	5.56 ± 0.06	5.68 ± 0.04	5.67 ± 0.09
Glo (g/dL)	1.45 ± 0.08	1.38 ± 0.04	1.56 ± 0.08	1.50 ± 0.08
Alb (g/dL)	4.17 ± 0.07	4.18 ± 0.05	4.12 ± 0.05	4.17 ± 0.06
BUN (mg/dL)	20.4 ± 1.5	19.4 ± 0.7	20.0 ± 0.5	19.2 ± 0.9
Cre (mg/dL)	0.22 ± 0.01	0.23 ± 0.02	0.22 ± 0.01	0.22 ± 0.01
TG (mg/dL)	136.2 ± 19.3	70.6 ± 14.2	101.4 ± 26.2	90.0 ± 22.2
TC (mg/dL)	66.2 ± 5.7	75.4 ± 1.1	74.6 ± 5.7	63.2 ± 2.6

*Females*				
ALP (U/L)	81.0 ± 2.5	80.2 ± 4.8	70.0 ± 5.5	68.8 ± 8.6
ALT (U/L)	29.2 ± 1.9	25.2 ± 1.1	24.8 ± 1.6	28.8 ± 1.4
AST (U/L)	127.5 ± 10.1	130.2 ± 24.0	130.0 ± 9.4	121.0 ± 8.8
T-bil (mg/dL)	<0.15	<0.15	<0.15	<0.15
D-bil (mg/dL)	<0.09	<0.09	<0.09	<0.09
TP (g/dL)	5.62 ± 0.02	6.27 ± 0.12	6.23 ± 0.19	6.60 ± 0.08
Glo (g/dL)	1.25 ± 0.03	1.47 ± 0.07	1.34 ± 0.10	1.57 ± 0.03^*∗*^
Alb (g/dL)	4.17 ± 0.07	4.80 ± 0.05	4.89 ± 0.16	5.03 ± 0.07
BUN (mg/dL)	20.4 ± 1.5	17.4 ± 0.6	16.2 ± 0.4	15.5 ± 0.5
Cre (mg/dL)	0.22 ± 0.01	0.28 ± 0.01	0.25 ± 0.01	0.22 ± 0.01
TG (mg/dL)	57.2 ± 10.2	77.4 ± 20.5	63.2 ± 7.8	86.5 ± 6.8
TC (mg/dL)	45.5 ± 1.7	46.4 ± 2.8	47.0 ± 5.8	64.8 ± 5.8^*∗*^

Data are expressed as the mean ± S.E.M., *n* = 5 animals/sex/group. ^*∗*^*p* < 0.05, significantly different from control group.

**Table 3:** Hematological parameters of NawaTab-treated rats in the subacute toxicity study.

Parameters	Control	NawaTab (mg/kg BW)		
		250	500	1000
*Males*				
WBC (×10^3^ cells/*μ*L)	4.02 ± 0.33	5.48 ± 0.52	5.04 ± 0.66	5.05 ± 0.56
NE (%)	5.64 ± 3.79	3.62 ± 1.57	3.37 ± 1.23	1.45 ± 0.09
LY (%)	82.44 ± 4.20	86.26 ± 1.83	85.03 ± 3.71	85.98 ± 1.20
MO (%)	10.42 ± 1.26	8.82 ± 0.64	10.77 ± 2.68	11.25 ± 1.23
EO (%)	1.08 ± 0.20	1.02 ± 0.15	0.57 ± 0.09	1.00 ± 0.30
BA (%)	0.42 ± 0.12	0.28 ± 0.02	0.27 ± 0.03	0.32 ± 0.05
RBC (×10^6^ cells/*μ*L)	7.89 ± 0.28	7.67 ± 0.11	7.92 ± 0.12	7.97 ± 0.18
HGB (g/dL)	14.76 ± 0.23	14.90 ± 0.32	14.83 ± 0.50	14.90 ± 0.31
HCT (%)	47.16 ± 1.42	47.04 ± 0.67	48.10 ± 1.79	47.82 ± 0.80
PLT (×10^3^ cells/*μ*L)	785.0 ± 69.4	789.0 ± 29.6	715.0 ± 47.5	680.0 ± 52.1
MCV (fL)	59.80 ± 0.89	61.30 ± 0.59	60.70 ± 1.95	60.10 ± 0.95
MCH (pg)	18.80 ± 0.48	19.40 ± 0.21	18.70 ± 0.59	18.70 ± 0.33
MCHC (g/dL)	31.34 ± 0.58	31.68 ± 0.28	30.87 ± 0.26	31.15 ± 0.30
RDW (%)	12.30 ± 1.08	12.02 ± 0.69	12.20 ± 0.15	11.15 ± 0.12

*Females*				
WBC (×10^3^ cells/*μ*L)	2.28 ± 0.53	2.61 ± 0.34	3.36 ± 0.51	3.51 ± 0.31
NE (%)	10.74 ± 3.51	9.20 ± 4.80	4.88 ± 2.14	2.70 ± 0.23
LY (%)	76.18 ± 4.55	76.70 ± 3.80	82.22 ± 2.48	85.85 ± 1.27
MO (%)	10.06 ± 1.13	12.50 ± 1.98	11.56 ± 0.36	10.35 ± 1.21
EO (%)	1.04 ± 0.25	1.15 ± 0.18	0.58 ± 0.08	0.52 ± 0.13
BA (%)	1.98 ± 0.94	0.45 ± 0.12	0.76 ± 0.24	0.58 ± 0.05
RBC (×10^6^ cells/*μ*L)	8.07 ± 0.12	7.52 ± 0.10	7.97 ± 0.17	7.99 ± 0.11
HGB (g/dL)	15.66 ± 0.27	14.52 ± 0.49	15.40 ± 0.32	15.72 ± 0.37
HCT (%)	46.86 ± 0.89	44.72 ± 1.08	46.84 ± 1.30	48.58 ± 0.74
PLT (×10^3^ cells/*μ*L)	596.0 ± 74.8	619.5 ± 78.2	720.6 ± 72.8	759.8 ± 31.1
MCV (fL)	58.10 ± 0.92	59.50 ± 0.73	58.70 ± 0.53	60.90 ± 1.20
MCH (pg)	19.40 ± 0.36	19.30 ± 0.49	19.30 ± 0.24	19.70 ± 0.44
MCHC (g/dL)	33.46 ± 0.58	32.48 ± 0.82	32.92 ± 0.47	32.38 ± 0.42
RDW (%)	11.16 ± 0.39	10.9 ± 0.64	11.28 ± 0.71	10.93 ± 0.28

Data are expressed as mean ± S.E.M, *n* = 5 animals/sex/group.

**Table 4:** Relative organ weight (g/100 g BW) of NawaTab-treated rats in the subacute toxicity study.

Parameters	Control	NawaTab (mg/kg BW)		
		250	500	1000
*Males*				
Thymus gland	0.20 ± 0.02	0.20 ± 0.02	0.18 ± 0.02	0.20 ± 0.02
Heart	0.30 ± 0.01	0.30 ± 0.00	0.31 ± 0.01	0.30 ± 0.00
Lung	0.38 ± 0.01	0.41 ± 0.01	0.41 ± 0.01	0.40 ± 0.01
Liver	3.41 ± 0.03	3.50 ± 0.11	3.50 ± 0.09	3.35 ± 0.10
Kidney	0.78 ± 0.02	0.80 ± 0.01	0.80 ± 0.01	0.79 ± 0.03
Spleen	0.21 ± 0.01	0.20 ± 0.01	0.20 ± 0.03	0.23 ± 0.01
Testis	1.03 ± 0.02	1.07 ± 0.03	1.13 ± 0.03	1.10 ± 0.03
Prostate gland	0.23 ± 0.01	0.21 ± 0.01	0.21 ± 0.01	0.21 ± 0.02
Epididymis	0.21 ± 0.02	0.22 ± 0.01	0.24 ± 0.00	0.24 ± 0.01
Vas deferens	0.05 ± 0.00	0.05 ± 0.00	0.05 ± 0.00	0.05 ± 0.00

*Females*				
Thymus gland	0.21 ± 0.01	0.22 ± 0.01	0.21 ± 0.01	0.22 ± 0.02
Heart	0.31 ± 0.01	0.32 ± 0.00	0.32 ± 0.01	0.32 ± 0.01
Lung	0.45 ± 0.01	0.46 ± 0.02	0.50 ± 0.05	0.43 ± 0.01
Liver	3.49 ± 0.07	3.66 ± 0.14	3.45 ± 0.07	3.64 ± 0.06
Kidney	0.83 ± 0.02	0.83 ± 0.04	0.83 ± 0.02	0.80 ± 0.02
Spleen	0.23 ± 0.01	0.24 ± 0.01	0.25 ± 0.01	0.23 ± 0.01
Ovary	0.06 ± 0.00	0.07 ± 0.01	0.07 ± 0.00	0.06 ± 0.00
Uterus	0.23 ± 0.04	0.22 ± 0.01	0.30 ± 0.05	0.30 ± 0.06

Data are expressed as the mean ± S.E.M., *n* = 5 animals/sex/group.

## Data Availability

The data used in this study are available upon reasonable request to the corresponding author.
